# Polysaccharides of *Scrophularia ningpoensis* Hemsl.: Extraction, Antioxidant, and Anti-Inflammatory Evaluation

**DOI:** 10.1155/2020/8899762

**Published:** 2020-12-15

**Authors:** Jian'an Wang, Lufen Huang, Qiang Ren, Yanjun Wang, Lirun Zhou, Yingjie Fu, Chunmei Sai, Shafii Shaibu Pella, Yingying Guo, Li-Na Gao

**Affiliations:** ^1^School of Pharmacy, Jining Medical University, Rizhao, Shandong 276826, China; ^2^Maternal and Child Health Care Family Planning Service Center, Ju Xian, Shandong 276500, China; ^3^Townsend Family Laboratories, Department of Psychiatry, The University of British Columbia, 2255 Wesbrook Mall, Vancouver BC V6T 1Z3, Canada

## Abstract

The roots of *Scrophularia ningpoensis* Hemsl. are a famous traditional Chinese medicinal herb and are also used as health food. However, information about polysaccharides from *S. ningpoensis* (SNPS) is very limited. We applied the ultrasonic-assisted extraction (UAE) process to extract SNPS. The UAE conditions were optimized using single-factor experiments and response surface analysis. Under the optimized conditions of ultrasonic power of 550 W, extraction time of 26 min, and extraction temperature at 50°C, the highest yield of 13.47% ± 1.63% was obtained, which was in accordance with the predicted value of 13.71%. In comparison with traditional hot water extraction, the optimized UAE method significantly increased the extraction yield with lower extraction temperature and shorter extraction time. Furthermore, the *in vitro* antioxidant evaluation showed that EC_50_ values of SNPS were 2.43 ± 0.21, 4.40 ± 0.35, and 0.56 ± 0.062 mg/mL for 2,2-diphenyl-1-picrylhydrazyl radical (DPPH) radical, hydroxyl free radical, and 2,2'-azinobis (3-ethylbenzothiazoline-6-sulfonic acid) (ABTS) radical scavenging assay, respectively. The anti-inflammatory potential of SNPS was detected in lipopolysaccharide (LPS) induced ICR mice. Real-time reverse transcription-polymerase chain reaction and enzyme-linked immunosorbent assay showed that SNPS significantly improved LPS-stimulated inflammatory response by decreasing mRNA and protein expression of interleukin (IL)-6 and tumour necrosis factor (TNF)-*α* in a dose-dependent manner. In conclusion, the extraction process of SNPS established in this study is reliable, and SNPS possesses potential antioxidant and anti-inflammatory activities, which will provide a theoretical basis for guiding the clinical application of *S. ningpoensis*.

## 1. Introduction

The roots of *Scrophularia ningpoensis* Hemsl. (*Radix Scrophulariae ningpoensis*) are a perennial plant which belongs to the Scrophulariaceae family. Its dried roots are known in China as Xuanshen, a traditional medicine that has been used as an antipyretic and for the treatment of sore throat and prurigo for over 1000 years [1, 2]. It has also been used as a health food. The main chemical substances of the roots of *S. ningpoensis* include iridoids, alkaloids, flavonoids, essential oils, and carbohydrates, which have been reported to possess anti-inflammatory actions [3–5]. Moreover, these plant-derived bioactive components are characterized by potential antioxidant activities [6, 7]. However, few studies focused on the activity of polysaccharides from *S. ningpoensis* (SNPS). Polysaccharides are among the most active substances found in nature, with a variety of biological activities, such as antitumour, antioxidation, anti-inflammation, and immune regulation, with relatively low toxicity and side effects [8–10].

The development of efficient extraction methods for polysaccharides is important. The main polysaccharides extraction methods include hot water extraction (HWE), ultrasonic-assisted extraction (UAE), enzyme-assisted extraction (EAE), and microwave-assisted extraction (MAE) [11–14]. The UAE method utilizes the mechanical and thermal action generated by the high-frequency oscillation of ultrasonic waves, which results in a sharp increase in the frequency and rate of movement of the molecules inside the materials, thereby causing the rupture of the tissues and cells and the release of the active ingredients. This method is widely used, simple to operate, and associated with high extraction efficiency [15, 16]. More importantly, UAE has potential advantages of protecting the structure and biological activity of active components [17–19].

In this study, the water-soluble polysaccharides from *S. ningpoensis* were extracted using the UAE method, and the extraction process was optimized by single-factor experiments and response surface method (RSM). After the deproteinization of the crude polysaccharides obtained from *S. ningpoensis*, the structure and monosaccharide compositions of SNPS were determined using Fourier transform infrared spectroscopy (FT-IR) and high-performance liquid chromatography (HPLC), respectively. To further investigated the antioxidant potential of SNPS, 2,2-diphenyl-1-picrylhydrazyl (DPPH) radical, hydroxyl free radical, and 2,2'-azinobis (3-ethylbenzothiazoline-6-sulfonic acid) (ABTS) radical scavenging tests were performed. Simultaneously, lipopolysaccharide (LPS) induced *in vivo* inflammatory mice model was used to detect the anti-inflammatory activities of SNPS. These results will provide a theoretical basis for the further development and utilization of polysaccharides from *S. ningpoensis*.

## 2. Materials and Methods

### 2.1. Chemicals and Reagents

The roots of *S. ningpoensis* were collected from the experimental field of Jining Medical University (Rizhao, Shandong, China) and identified by Jian'an Wang (Medical Botanist, Jining Medical University). Monosaccharide standards D-glucose (D-Glc), D-galactose (D-Gal), D-arabinose (D-Ara), L-fucose (L-Fuc), D-mannose (D-Man), L-rhamnose (L-Rha), D-fructose (D-Fru), D-xylose (D-Xyl), D-GlcA, and D-GalA were purchased from Sigma-Aldrich (St. Louis, MO, USA). 2,2-Diphenyl-1-picrylhydrazyl (DPPH) free radical was purchased from Sigma-Aldrich (St. Louis, MO, USA). 2,2'-azinobis (3-ethylbenzothiazoline-6-sulfonic acid) (ABTS) free radical was purchased from the Shanghai Yuanye Biotechnology Co. Ltd. (Shanghai, China). ImProm-II Reverse Transcription System was obtained from Promega Corporation (Madison, WI, USA). IL-6 Mouse ELISA Kit was purchased from Invitrogen (Carlsbad, CA, USA). TNF-*α* Mouse ELISA Kit was obtained from PeproTech (Rocky Hill, NJ, USA). UNIQ-10 column Trizol total RNA extraction kit was obtained from Sangon Biological Engineering Technology and Services Co., Ltd. (Shanghai, China). FastStart Universal SYBR Green Master (ROX) kit was purchased from Roche (Mannheim, Germany). All chemical reagents were of analytical grade, and the deionized water was used in the whole experiment.

### 2.2. Pretreatment of the Roots of S. Ningpoensis

After washing the roots of *S. ningpo*ensis, they were drained and then dried under vacuum at 65°C. Root powder was obtained after pulverizing and sifting the dried roots with a 40-mesh sieve. Subsequently, the dried powder was mixed with acetone (at a ratio of 1 : 5) to remove fat-soluble substances by uniformly stirring for 24 h. The precipitate was concentrated and lyophilized (40°C, 12 h) to obtain the pretreated root sample.

### 2.3. Ultrasonic-Assisted Extraction Process

UAE was performed according to the described procedure of Jian-Hua Xie with slight modifications [20]. In detail, a total of 5.0 g of the pretreated *S. ningpoensis* powder was extracted by adding 150 mL deionized water. The reaction system was placed in an ultrasonic cell pulverizer (Xinyi 1200E, Ningbo Xinyi Ultrasonic Equipment Co., Ltd., Ningbo, China) with a low constant temperature tank (DC-8006, Ningbo Xinyi Ultrasonic Equipment Co., Ltd., Ningbo, China). The ultrasonic power was set to 285, 380, 475, 570, and 665 W, respectively. The extraction temperature was set to 30, 40, 50, 60, and 70°C, respectively. The extraction time was set to 5, 15, 25, 35, and 45 min, respectively. The solutions obtained after the reactions were diluted with a quadruple volume of absolute ethanol to a final alcohol content of 80%, and the mixtures were stored at 4°C overnight and then centrifuged at 3500 rpm for 15 min. The precipitates were freeze-dried to obtain the crude polysaccharides. The resulting crude SNPS were redissolved in deionized water, deproteinized according to the method reported by Sevag et al. [20], and lyophilized to obtain purified SNPS for subsequent analysis.

The extract yield of SNPS was determined by the phenol sulfuric acid method with minor modification [21] and calculated according to the following formula:

(1) $$\begin{array}{c} \text{Extraction yield }\left(\%\right)\;=\frac{C\times V}{W}\times 100\%, \end{array}$$

where *C* (mg/mL) is the concentration of the SNPS solution, *V* is the dilution factor, and W is the dry weight of the pretreated powder.

### 2.4. Optimization of Extraction Process

Based on the results of monofactor tests, RSM was further used to optimize the extraction parameters. BBD was applied to determine the effect of extract temperature (*X*_1_, °C), ultrasonic power (*X*_2_, *W*), and extract ultrasonic time (*X*_3_, min) at three levels on the extraction yield of SNPS. As shown in Table 1, the above 3 factors were designed at three levels respectively, and −1, 0, and 1 represented the low, medium, and high levels of the independent variables, respectively.

Based on experiment results from BBD, a second-order polynomial model was performed as follows:

(2) $$\begin{array}{c} Y=A_{o}+\sum_{i=1}^{3}A_{i}X_{i}+\sum_{i=1}^{3}A_{ii}X_{i}^{2}+\sum_{i=1}^{2}\sum_{i=j+1}^{3}A_{ij}X_{i}X_{j}, \end{array}$$

where *Y* is the response variable (extraction yield of SNPS); *A*_0_ is a constant, *Ai* is the linear coefficient, *Aii* is the quadratic coefficient, and *Aij* is the interaction coefficient. *Xi* and *Xj* are independent variables (*i* ≠ *j*).

### 2.5. Monosaccharide Component Analysis

The monosaccharide component was determined by HPLC (Waters1525, Waters Co., USA). A total of 2.0 mg SNPS sample was mixed with 1 mL trifluoroacetic acid (TFA, 2 mol/L) and then hydrolyzed in an oven at 121°C for 2 h. Subsequently, the volume was adjusted to 50 mL with water, and the mixture was filtered with a 0.45 *μ*m microporous membrane for HPLC analysis. All standard sugars were prepared according to the same method. The sample and standards were analyzed by ion chromatography using ICS-5000 ion chromatograph (Dean Inc., USA) with a pulsed amperometric detector (PAD) as previously reported [22], with some modifications. An UltrahydrogelTM Linear column (300 mm × 7.8 mm) was used with a flow rate of 0.5 mL/min. The detailed liquid phase was shown in Table 2.

### 2.6. FT-IR Analysis of SNPS

A total of 1.0 mg of SNPS was mixed with potassium bromide for tableting, and the dried polysaccharides were subjected to 4000-400 cm^−1^ scanning using FT-IR (IR Tracer-21, Shimadzu Corporation, Tokyo, Japan).

### 2.7. DPPH Radical Scavenging Activity of SNPS

The DPPH radical scavenging activity of SNPS was evaluated as previously reported [23], with slight modifications. Briefly, SNPS solutions were diluted with DPPH-ethanol solution and incubated at room temperature in the dark for 30 min. The DPPH solution was replaced with anhydrous ethanol in the control tube, and the polysaccharides solution was replaced with distilled water in the blank tube. The absorbance of each solution was measured at a wavelength of 517 nm and ascorbic acid (Vitamin *c*, Vc) was used as a positive control. The scavenging rate was calculated according to the following formula:

(3) $$\begin{array}{c} \text{Scavenging activity }\left(\%\right)=\left(1-\frac{A_{S}-A_{C}}{A_{b}}\right)\times 100, \end{array}$$

where *A*_*s*_ was the absorbance of DPPH-ethanol solution, deionized water and SNPS samples, *A*_*c*_ was the absorbance of SNPS solution with equivolumetric anhydrous ethanol instead of DPPH, *A*_*b*_ was the absorbance of DPPH solution with equivolumetric deionized water (without SNPS).

### 2.8. Hydroxyl Free Radical Scavenging Rate of SNPS

The hydroxyl radicals scavenging activity of SNPS was determined according to the method reported by Li with a slight modification [24]. In short, SNPS was added sequentially with 2.0 mL ferrous sulfate solution (9 mmol/L, FeSO_4_), 2.0 mL salicylic acid in ethanol (9 mmol/L, C_7_H_6_O_3_), and 2.0 mL hydrogen peroxide solution (9 mmol/L, H_2_O_2_). The mixtures were shaken well and incubated at 37°C for 30 min. Deionized water was used as a reference and the absorbance was measured at a wavelength of 510 nm. Vc of the same mass concentration was used as a positive control. The scavenging rate was calculated according to equation (3), where As was the absorbance of the samples in the FeSO_4_–C_7_H_6_O_3_ reaction system, Ac was the absorbance of the samples in deionized water, and Ab was the absorbance of the FeSO_4_–C_7_H_6_O_3_ reaction system with deionized water.

### 2.9. ABTS Free Radical Scavenging Rate of SNPS

The ABTS radical scavenging activity of SNPS was determined using a modified method, according to Re et al. [25]. Equivalent amounts of 7 mmol/L ABTS solution and 2.45 mmol/L potassium persulfate solution were mixed and incubated at room temperature in the dark for 12–16 h to prepare the ABTS^ + ^stock solution. Then the ABTS^ + ^assay solution was obtained by diluting the stock solution with distilled water. Different concentrations of SNPS samples were mixed with ABTS^ + ^assay solution followed by incubation at room temperature in the dark for 60 min. The absorbance was measured immediately at 734 nm. Vc was used as a positive control. The ABTS radical scavenging activity was calculated using equation (3), with ABTS solution instead of DPPH.

### 2.10. In Vivo Anti-Inflammatory Evaluation of SNPS

Male-specific pathogen-free (SPF) grade imprinting control region (ICR) mice (18–22 g) were purchased from Qingdao Daren Fortune Animal Technology Co., Ltd (Qingdao, China). All mice were housed under controlled environmental conditions (temperature, 22 ± 1°C; relative humidity, 50 ± 5%; light/dark cycle, 12 h). The animal experiments were conducted according to the NIH Guide for the Care and Use of Laboratory Animals and the protocol was approved by the Ethics Committee of Jining Medical University.

After adaptation for 7 days, a total of 48 mice were randomly assigned into 6 groups (*n* = 8): (1) Normal control group; (2) Model group; (3) Positive group (aspirin 10 mg/kg); (4–6) SNPS (100, 200 or 400 mg/kg, respectively). SNPS was dissolved in sterile pyrogen-free saline solution. The mice in the normal control group and model group were gastrointestinally treated with 0.9% saline for 4 days (once per day). Mice in the positive group and the SNPS group were treated with aspirin and corresponding dosages of SNPS, respectively, for 4 days, once per day. Thirty minutes after the final administration, the mice (in addition to the control group) were intraperitoneally injected with LPS (1 mg/kg). The mice in the control group were treated with equal volumes of saline. After 90 min, all mice were sacrificed by diethyl ether asphyxiation. The blood was collected, and serum was prepared and stored at −20°C for the detection of protein levels of TNF-*α* and IL-6. Simultaneously, the liver tissues were harvested and weighed; the liver tissues were used for the determination of the mRNA expression.

### 2.11. Real-Time RT-PCR Assay

The mRNA expressions of IL-6 and TNF-*α* in the mouse liver were detected by real-time RT-PCR. In brief, the liver tissues (approximately 50 mg) were homogenized in 500 *μ*L Trizol, and the total RNA was isolated using a Sangon UNIQ-10 column Trizol total extraction kit. Then, reverse transcriptions were performed using the ImProm-II Reverse Transcription System cDNA synthesis kit according to the manufacturer's instructions. The real-time RT-PCR oligonucleotide primers used for the mouse IL-6, TNF-*α,* and *β*-actin (an internal control) are listed in Table 3. The reaction processes were performed as previously described [26].

### 2.12. Analysis for Serum IL-6 and TNF-*α* Expression

The serum concentrations of TNF-*α* and IL-6 were detected according to the commercial ELISA kits. The serum samples for TNF-*α* and IL-6 were assayed at a 100-fold and 50-fold dilution in the assay buffer, respectively.

### 2.13. Statistical Analyses

Statistical analysis was performed using the Origin 8.0 software (Northampton, MA, USA). In vitro, data were expressed as the means ± S.D. from three independent experiments. In vivo, data were expressed as the means ± S.E. Differences between groups were analyzed by one-way analysis of variance (ANOVA). A *P*-value less than 0.05 was considered statistically significant.

## 3. Results and Discussion

### 3.1. Effect of Ultrasonic Power on the Yield of SNPS

**Figure 1:**
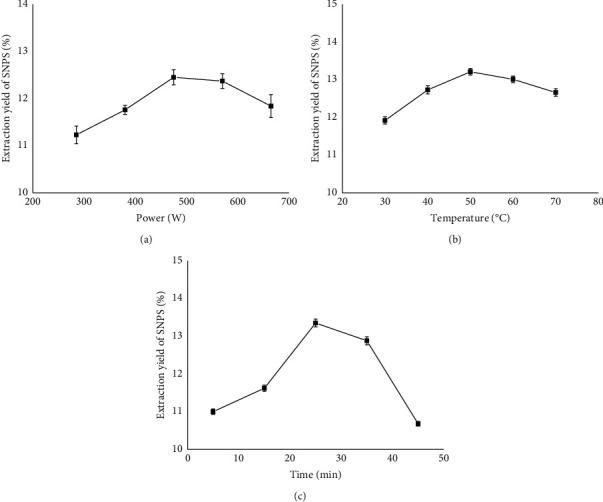
Effect of different extraction parameters on the yield of SNPS. (a) Ultrasonic power (W). (b) Extraction temperature (°C). (c) Extraction time (min).

In this study, the UAE method was applied to extract SNPS from the roots of *S. ningpoensis*. The extraction power, temperature, and time were evaluated, and the results are shown in Figure 1. To examine the effect of the ultrasonic power on the extraction efficiency, the ratio of material to liquid, extraction temperature, and extraction time was set as 1 : 30 g/mL, 50 °C and 25 min, respectively. As shown in Figure 1(a), with an ultrasonic power of 475 W, the extraction efficiency reached its maximum of 12.45% ± 0.16%. Subsequently, however, the extraction yield decreased with a further increase of ultrasonic power. The ultrasonic power, therefore, had a significant influence on the extraction yield. We speculated that higher ultrasonic power would induce the degradation of polysaccharides [27]. Therefore, 475 W was chosen as the optimal extraction power for subsequent optimization experiments.

### 3.2. Effect of Ultrasonic Temperature on the Yield of SNPS

A total of 5.0 g of acetone-extracted *S. ningpoensis* powder was weighed accurately. The ratio of material to liquid, ultrasonic power, and extraction time was set as 1 : 30 g/mL, 475 W, and 25 min, respectively, in order to detect the effect of temperature on the extraction efficiency. When the temperature increased, the vapor pressure made more solvent vapors enter the bubble cavity and induce the collapse of the cell wall. Thus, it made the solvent penetrate deeply into the sample matrix, resulting in higher extraction efficiency. As shown in Figure 1(b), a maximum yield of 13.21% ± 0.087% was observed at 50°C. The extraction efficiency decreased at higher temperatures, likely due to the increased dissolution of impurities [28, 29].

### 3.3. Effect of Extraction Time on the Yield of SNPS

The effect of the ultrasonic time on the yield of SNPS was investigated from 5 to 45 min, with other extraction conditions fixed as follows: the ratio of material to liquid, 1 : 30 g/mL; ultrasonic power, 475 W; and temperature, 50°C. As shown in Figure 1(c), the yield of SNPS increased from 10.90% ± 0.073% to 13.35% ± 0.10% from 5 to 25 min, respectively. The yield then decreased with increasing extraction time, likely due to the ultrasonic cavitation energy, which generated many cavities on the external surface of the plant's cell wall, thus accelerating the release of the bioactive constituents into the liquid [30]. A previous study showed that excessive ultrasonication durations cause polysaccharide degradation [31]. According to the results from the single-factor studies, extraction power, temperature, and time significantly affected the yield of SNPS. Therefore, we further optimized these three parameters.

### 3.4. Optimization of SNPS Extraction by BBD

#### 3.4.1. Fitting the Model

The effect of the extraction temperature (*X*_1_), ultrasonic power (*X*_2_), and ultrasonic time (*X*_3_) on the extraction yield of SNPS was evaluated at three levels using BBD. The results are shown in Table 4. The yield of SNPS ranged from 10.56% ± 0.69% to 13.71% ± 1.63%. Experiment 14 (extraction temperature, 50°C; extraction power, 475 W; extraction time, 25 min) produced the highest yield, while experiment 5 (extraction temperature, 30°C; extraction power, 475 W; extraction time, 15 min) produced the lowest yield. The following second-order polynomial was obtained by multiple regression analysis:

(4) $$\begin{array}{c} \text{Yield}\%=13.38+0.071X_{1}+0.43X_{2}+0.27X_{3}+0.20X_{1}X_{2}-0.38\times 1\;\times 3+0.077X_{2}X_{3}-0.82{\left(X_{1}\right)}^{2}-0.57{\left(X_{2}\right)}^{2}-1.10{\left(X_{3}\right)}^{2}. \end{array}$$

According to the ANOVA of the quadratic regression model (Table 5), the values of determination coefficient *R*^2^ was 0.954, indicating that 95.4% of the variables can be explained by the fitting model. The adjusted determination coefficient *R*^2^ (*R*^2^_adj_, 0.895) indicated a relatively high degree of correlation between the experimental and predicted values. The coefficient of variation (C.V.%) was 2.47, indicating the reliability of the experimental values. The F-value (2.26) and *P*-value (0.224) of the lack-of-fit factor test represented there was no lack-of-fit factor in this model.

Furthermore, the linear coefficients (*X*_2_ and *X*_3_), the quadratic term coefficients (*X*_1_^2^, *X*_2_^2^, and *X*_3_^2^), and the interaction coefficient (*X*_1_*X*_3_) affected the yield of SNPS highly significantly (*P* < 0.05 or *P* < 0.01), while the other coefficients were not significant (*P* > 0.05). The F-value showed that the extraction yield was influenced by the three ultrasonic extraction parameters, in the following order: power > time > temperature; the interaction effects were in the following order: *X*1*X*3>*X*1*X*2>*X*2*X*3. Therefore, the regression equation can be applied to demonstrate the real relationship between the extraction yield of SNPS and variables.

#### 3.4.2. Diagnosis of Model Adequacy

**Figure 2:**
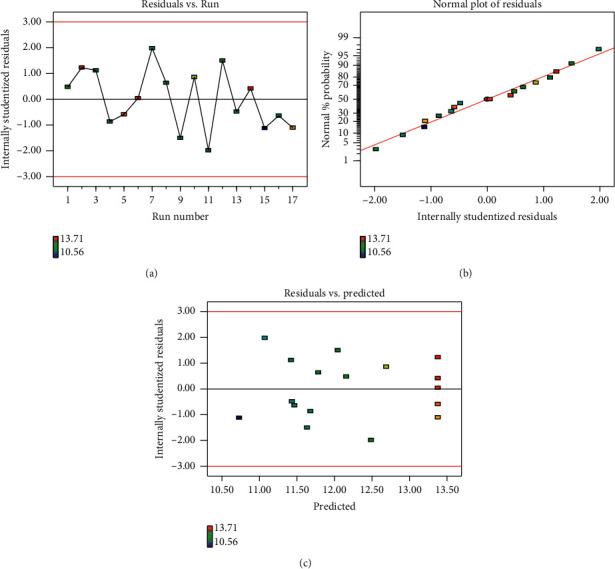
Diagnostic plots for the model adequacy. (a) The plot of internally studentized residuals vs. the actual run. (b) Normal probability plot of the studentized residuals. (c) Residuals vs. predicted plot graph.

Model adequacy is very vital for checking whether the established model would give a sufficient approximation to the actual values.  Figure 2(a) shows the internally studentized residuals versus actual runs; all data points lay within the acceptable limits (±2). As shown in Figure 2(b), the data points lay almost close to the straight line, indicating that the normality assumption was satisfied. Meanwhile, the plot of internally studentized residuals versus predicted response values (Figure 2(c)) showed that the residuals randomly scattered within the range of −3 and + 3 in the *y*-axis, suggesting that the predicted response was within the acceptable limits. Therefore, these data implied that the present model was reliable.

#### 3.4.3. Response Surface Analysis

**Figure 3:**
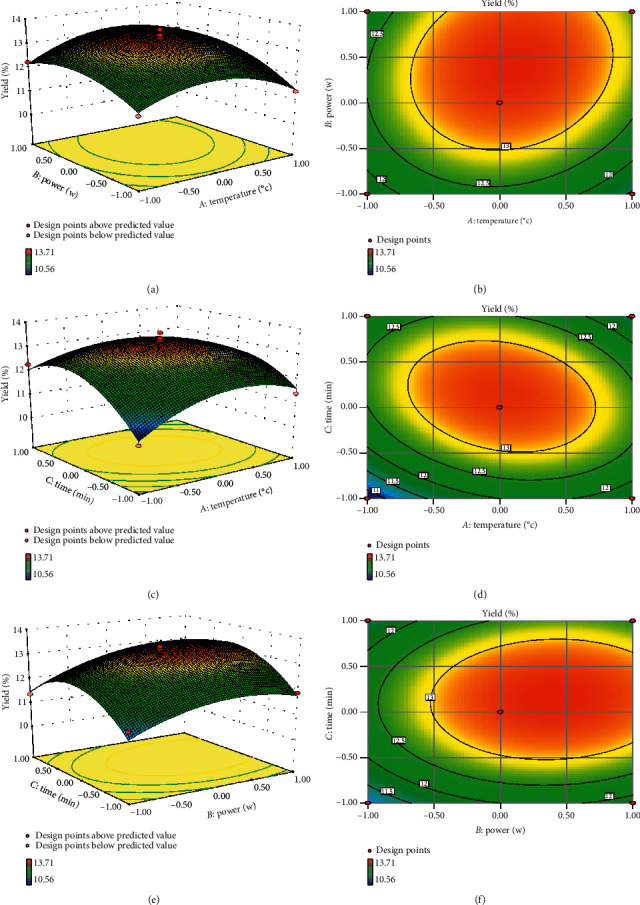
Interactive effects of ultrasonic power, ultrasonic temperature, and ultrasonic time on the yield of SNPS. (a and b) Response surface and contour plots of the effect of ultrasonic power and temperature on the extraction rate of SNPS; (c and d) Response surface and contour plots of the effect of ultrasonic time and temperature on the extraction rate of SNPS; (e and f) Response surface and contour plots of the effect of ultrasonic power and time on the extraction rate of SNPS.

A response surface analysis was performed, including the extraction yield, extraction conditions, and level of each extraction condition; this map highlights the influence of each extraction condition on the response value [32]. The values of the extraction conditions, and the interactions between the extraction conditions under optimal conditions, can be revealed by a contour map [33]. In the present study, the 3D response surface and 2D contour plots showed the relationship between the extraction temperature (*X*_1_), extraction power (*X*_2_), extraction time (*X*_3_), and extraction yield of SNPS (Figure 3). Figures 3(a) and 3(b) illustrate the effect of extraction temperature and extraction power on the yield when the extraction time was set as 25 min. The extract yield of SNPS initially increased when the extraction temperature and power increased in the range of 30–51.24°C and 285–550.63 W, respectively. However, with the above extract temperature of 51.24°C and ultrasonic power 550.63 W, a decreased extract yield was observed. Thus, the highest yield was obtained with ultrasonic power of 550.63 W and extract temperature of 51.24°C. In addition, the curved surface of the extraction power was steeper than that of the extraction temperature, suggesting that extraction power influenced the yield more significantly than that of the extraction temperature.

Figures 3(c) and 3(d) exhibit the effects of the extraction temperature and extraction time on the yield of SNPS. When fixing the extraction power at 475 (W), an obvious increase in the yield was observed with the extraction temperature increasing from 30°C to 51.24°C, and the extraction time increasing from 5 min to 26.28 min. However, the extraction yield decreased, followed by prolonging the ultrasonic time and temperature in the range of 26.28–45 min and 51.24–70°C, respectively. The extraction yield of SNPS was the highest when the ultrasonic time was 26.28 min, and the temperature was 51.24°C. Statistically, the interaction effect between extraction temperature and time was significant (*P*=0.0389, as shown in Table 5).

Figures 3(e) and 3(f) illustrate the interactions between the extraction power and extraction time on the yield of SNPS when fixing the temperature at 50°C. When the ultrasonic power and time varied within the range of 285–550.63 (W) and 5–26.28 min, respectively, the extract yield gradually increased. However, the yield then decreased with increasing extraction time from 26.28 min to 45 min or ultrasonic power from 550.63 W to 665 (W). It indicated that SNPS was more likely to be degraded at a high extraction power. The yield of SNPS reached the maximum with ultrasonic power of 550.63 W and ultrasonic time of 26.28 min.

#### 3.4.4. Experimental Verification of the Regression Model

The optimal extraction conditions calculated from the model are as follows: ultrasonic time of 26.28 min, the ultrasonic temperature at 51.24°C, and ultrasonic power of 550.63 W. Under this condition, the yield of SNPS reached to 13.48%. To easy control the extraction parameters during actual operation, the optimal extraction conditions were corrected with a slight modification of ultrasonic time of 26 min, ultrasonic power of 550 W, and temperature at 50°C. Under these conditions, the actual extraction yield of SNPS was 13.47% ± 1.63% (*n* = 3), and the yield rate of purified polysaccharides extract 2.15% ± 0.12% (*n* = 3), suggesting that the parameters of the ultrasonic extraction condition optimized by the RSM are accurate and reliable.

### 3.5. Monosaccharide Composition and FT-IR Characterization of SNPS

**Figure 4:**
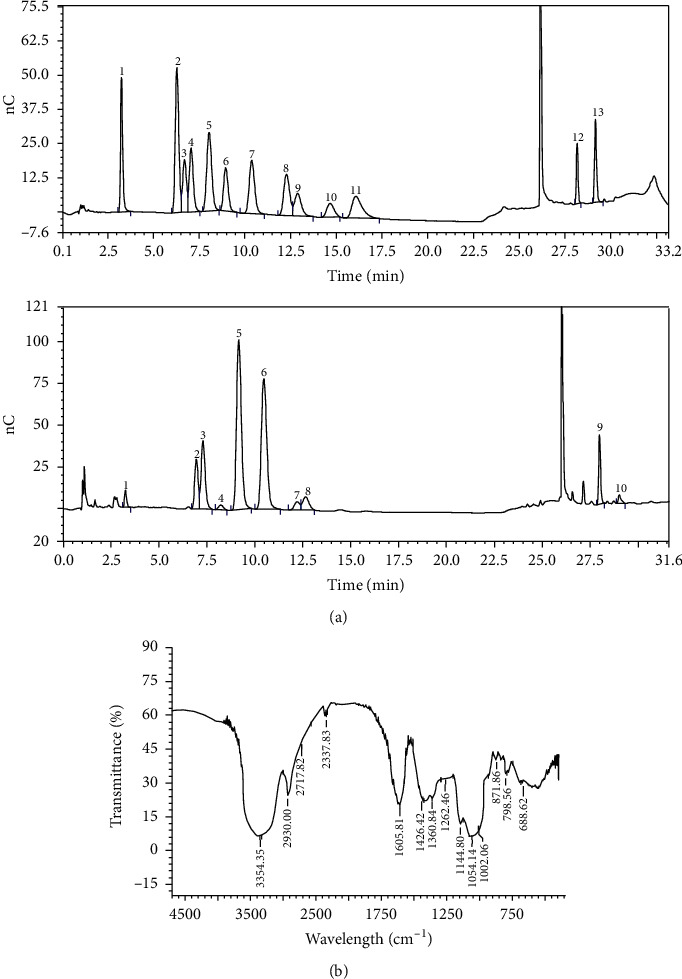
Characterization of SNPS. (a) HPLC analysis of monosaccharide components of SNPS (1. Fucose; 2. Rhamnose; 3. Arabinose; 4. Galactosamine; 5. Galactose; 6. Glucose; 7. Xylose; 8. Mannose; 9. Galacturonic acid; 10. Glucuronic acid). (b) FT-IR analysis of SNPS.

The total carbohydrate content of SNPS was 91.53% as determined by the phenol sulfuric acid method. Total protein content of 1.086% was determined by the Coomassie Brilliant Blue method. The monosaccharide composition determined by HPLC is shown in Figure 4(a). SNPS consisted of 1.30% fucose, 6.34% rhamnose, 9.95% arabinose, 0.82% aminogalactose, 29.98% galactose, 25.99% glucose, 1.56% xylose, 2.97% mannose, 4.86% galacturonic acid, and 0.90% glucuronic acid.

As shown in Figure 4(b), SNPS presents the typical absorption peaks of polysaccharides in the range of 4500-500 cm^−1^. The strong and broad signal at 3354 cm^−1^ corresponds to the O–H stretching vibration in the constituent sugar residues [34]. The absorption peak at 2930 cm^−1^ represents a stretching vibration of the C–H of the sugar ring. The absorption peaks at 1426 cm^−1^ and 1360 cm^−1^ were assigned to C–O stretching vibrations. The strong absorptions at 1140 cm^−1^, 1064 cm^−1^, and 1002 cm^−1^ indicate the vibrations of C–O–C and C–O–H bond [35].

### 3.6. Antioxidant Activities of SNPS

**Figure 5:**
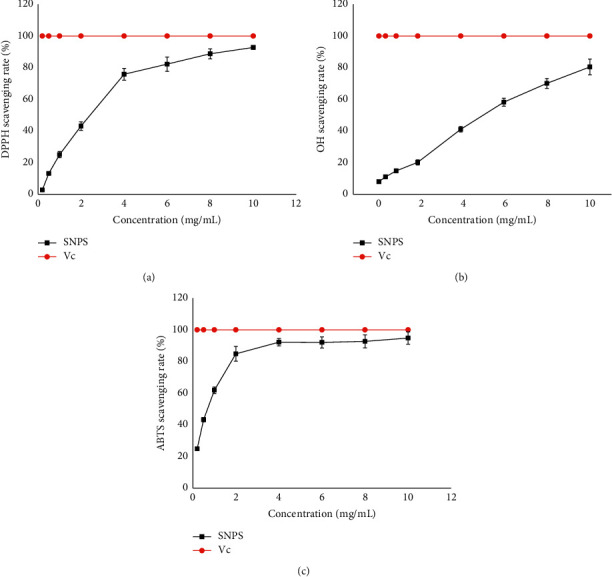
Antioxidant activities of SNPS. (a) Scavenging of DPPH radical. (b) Scavenging of OH free radical. (c) Scavenging of ABTS radical. Data are presented as the mean means ± S.D., *n* = 4. Vc was used as a positive control.

DPPH is a stable free radical that can accept an electron or hydrogen radical, forming a stable diamagnetic molecule with decreased absorbance at 517 nm. DPPH has been widely used to investigate the radical scavenging activity of natural polysaccharides [36]. As shown in Figures 5(a) and 5(b), SNPS and the positive control (Vc) exhibited obvious and concentration-dependent radical scavenging activity using DPPH radical and hydroxyl radical assay. The EC_50_ value of SNPS on DPPH and OH was 2.43 ± 0.21 mg/mL and 4.40 ± 0.35 mg/mL, respectively.

The ABTS assay is a decolorization assay applicable for both lipophilic and hydrophilic antioxidants at different _P_H levels [37]. The ABTS radical scavenging activities of SNPS and Vc are shown in Figure 5(c). Significant and dose-dependent ABTS scavenging activity was observed, even at SNPS concentrations <1.0 mg/mL. The maximum scavenging rate reached as high as 94.79% and the EC_50_ value was 0.56 ± 0.062 mg/mL. These data indicated that SNPS has a strong antioxidant activity.

### 3.7. Anti-Inflammatory Activities of SNPS

**Figure 6:**
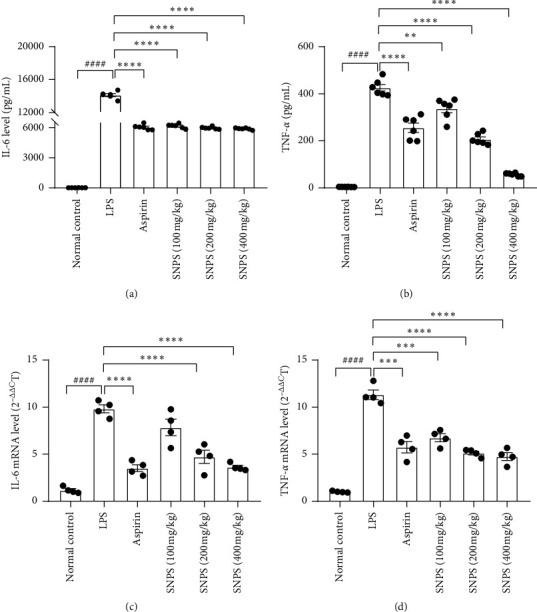
Anti-inflammatory activities of SNPS on LPS-induced mice. Mice were treated with SNPS (100, 200, and 400 mg/kg), aspirin (10 mg/kg), or saline for 4 days (once per day) and stimulated with LPS (1 mg/kg) for 90 min. The protein expression of (a) IL-6 and (b) in serum were determined by ELISA. Values are means ± S.E. (n = 6). The mRNA expression of IL-6 (c) and TNF-*α* (d) in the liver was measured using real-time RT-PCR. Values are means ± S.E. ((*n*) = 4). The *β*-actin was used as an internal control for real-time RT-PCR. Aspirin was used as a positive control. The significant difference compared with the normal control group, ^####^*P* < 0.0001. The significant difference compared with LPS-treated alone, ^*∗∗*^*P* < 0.01, ^*∗∗∗*^*P* < 0.001, ^*∗∗∗∗*^*P* < 0.0001.

LPS is a prototypical bacterial endotoxin that can initiate a variety of inflammatory responses, resulting in the production of various proinflammatory cytokines, which causes liver damage *in vitro* [38]. In the early inflammatory response, IL-6 and TNF-*α* play a vital role in evoking inflammation. In the present study, we measured the mRNA expression of IL-6 and TNF-*α* to investigate the anti-inflammatory action of SNPS to further confirm the effectiveness of SNPS extraction. As displayed in Figures 6(a) and 6(b), LPS injection induced a burst of IL-6 and TNF-*α* secretion in the serum (*P* < 0.01) in comparison with the normal control group. However, the treatment of SNPS or aspirin significantly suppressed the overproduction of IL-6 and TNF-*α* (*P* < 0.01). Additionally, the anti-inflammatory effect was more pronounced in the expression of TNF-*α*, indicating that SNPS may be effective for early inflammation treatment. The effect of SNPS on the expression of IL-6 and TNF-*α* mRNA in the liver was also investigated. As displayed in Figure 6(c) and 6(d), aspirin or SNPS treatment could effectively alleviate LPS-induced IL-6 and TNF-*α* mRNA increment (*P* < 0.01).

## 4. Conclusion

In the present study, an optimized UAE method was designed to extract polysaccharides from the roots of *S. ningpoensis*. Single-factor studies and RSM were employed to optimize the extraction conditions. At an optimal condition that ultrasonic power of 550 W, extraction temperature of 50°C, and extraction time of 26 min, the highest yield of SNPS (13.47% ± 1.63%) was obtained. Compared with the traditional hot water extraction method (the yield was 11.5%), the UAE method we applied significantly decreased extraction temperature and time and increased the extraction yield. The monosaccharide composition of SNPS analyzed by HPLC showed that SNPS was mainly composed of galactose and glucose, with lower levels of other monosaccharides. The FT-IR results illustrated that SNPS consisted primarily of carbohydrate substances, with no observed impurities. Furthermore, antioxidant and anti-inflammatory evaluations revealed that SNPS exhibited good antioxidant and anti-inflammatory activities in a dose-dependent manner. In conclusion, we proposed a reliable extraction process of polysaccharides and the resulting SNPS possesses potential biological activities, which will provide a theoretical basis for guiding the clinical application of *S. ningpoensis*.

## Figures and Tables

**Table 1:** Coded and uncoded levels of independent variables used for BBD.

Independent variable	Coded levels		
	−1	0	1
Ultrasonic temperature (*X*_1_) (°C)	30	50	70
Ultrasonic power (*X*_2_) (W)	285	475	665
Ultrasonic time (*X*_3_) (min)	15	25	35

**Table 2:** Mobile phase and gradient for monosaccharide composition analysis.

Time (min)	Water (%)	NaOH (250 mM, %)	NaAc (1 M, %)
0	97.4	2.6	0
21	97.4	2.6	0
21.1	92.4	2.6	5
30	77.4	2.6	20
30.1	20	80	0
50	20	80	0

**Table 3:** The real-time RT-PCR oligonucleotide primers.

Gene	Primer	Sequence (5'-3')	PCR product (bp)
*β*-actin	Forward	TGTTACCAACTGGGACGACA	165
(NM_007393.3)	Reverse	GGGGTGTTGAAGGTCTCAAA	—
IL-6	Forward	TCCAGTTGCCTTCTTGGGAC	140
(NM_031168.1)	Reverse	GTGTAATTAAGCCTCCGACTTG	—
TNF-*α*	Forward	TAGCCAGGAGGGAGAACAGA	127
(NM_013693.2)	Reverse	TTTTCTGGAGGGAGATGTGG	—

**Table 4:** Box-Behnken design with independent variables and response values.

Run	Independent variables			Response	
	*X*_1_ (°C)	*X*_2_ (W)	*X*_3_ (min)	Experimental yield (%)	Predicted yield (%)
1	−1 (30)	−1 (285)	0 (25)	11.55 ± 0.99	11.68
2	1 (70)	−1 (285)	0 (25)	11.36 ± 1.01	11.43
3	−1 (30)	1 (665)	0 (25)	12.23 ± 1.11	12.16
4	1 (70)	1 (665)	0 (25)	12.82 ± 0.87	12.69
5	−1 (30)	0 (745)	−1 (15)	10.56 ± 0.69	10.73
6	1 (70)	0 (745)	−1 (15)	11.41 ± 0.88	11.64
7	−1 (30)	0 (745)	1 (35)	12.27 ± 1.00	12.04
8	1 (70)	0 (745)	1 (35)	11.59 ± 0.93	11.42
9	0 (50)	−1 (285)	−1 (15)	11.37 ± 1.12	11.07
10	0 (50)	1 (665)	−1 (15)	11.88 ± 0.77	11.78
11	0 (50)	−1 (285)	1 (35)	11.37 ± 0.83	11.47
12	0 (50)	1 (665)	1 (35)	12.19 ± 1.03	12.49
13	0 (50)	0 (745)	0 (25)	13.22 ± 1.10	13.38
14	0 (50)	0 (745)	0 (25)	13.71 ± 1.63	13.38
15	0 (50)	0 (745)	0 (25)	13.39 ± 0.92	13.38
16	0 (50)	0 (745)	0 (25)	13.49 ± 0.88	13.38
17	0 (50)	0 (745)	0 (25)	13.08 ± 1.12	13.378

Data show mean ± SD (*n* = 3).

**Table 5:** Analysis of variance (ANOVA) of the quadratic model and lack-of-fit.

Source	Sum of squares	Df	Mean square	*F*-value	*P* Value	Significance
Model	13.22	9	1.47	16.13	0.00007	^*∗∗∗*^<0.0001
*X*_1_	0.041	1	0.041	0.45	0.5256	—
*X*_2_	1.51	1	1.51	16.53	0.0048	^*∗∗*^<0.01
*X*_3_	0.60	1	0.60	6.64	0.0366	^*∗*^<0.05
*X*_1_ *X*_2_	0.15	1	0.15	1.67	0.2372	—
*X*_1_ *X*_3_	0.59	1	0.59	6.43	0.0389	^*∗*^<0.05
*X*_2_ *X*_3_	0.024	1	0.024	0.26	0.6233	—
*X*_1_^2^	2.81	1	2.81	30.83	0.0009	^*∗∗∗*^<0.0001
*X*_2_^2^	1.38	1	1.38	15.10	0.0060	^*∗∗*^<0.01
*X*_3_^2^	5.13	1	5.13	56.36	0.0001	^*∗∗∗*^<0.0001
Residual	0.64	7	0.091	—	—	—
Lack-of-fit	0.40	3	0.13	2.26	0.224	—
Pure error	0.24	4	0.059	—	—	—
Cor total	13.86	16	—	—	—	—
*R* ^2^	0.954	—	—	—	—	—
Adj. *R*^2^	0.895	—	—	—	—	—
Adeq. Precision	11.447	—	—	—	—	—

C.V.% = 2.47, ^*∗*^significant at 0.05 level; ^*∗∗*^significant at 0.01 level; ^*∗∗∗*^ significant at 0.001 level.

## Data Availability

The datasets used in and/or analyzed during the current study available from the corresponding author on reasonable request.
